# How do frontline staff use patient experience data for service improvement? Findings from an ethnographic case study evaluation

**DOI:** 10.1177/1355819619888675

**Published:** 2020-02-14

**Authors:** Louise Locock, Catherine Montgomery, Stephen Parkin, Alison Chisholm, Jennifer Bostock, Sue Dopson, Melanie Gager, Elizabeth Gibbons, Chris Graham, Jenny King, Angela Martin, John Powell, Sue Ziebland

**Affiliations:** 1 Professor of Health Services Research, Health Services Research Unit, University of Aberdeen, UK; 2 Senior Researcher, Nuffield Department of Primary Care Health Sciences, University of Oxford, UK; 3 Research Fellow, National Addiction Centre, Institute of Psychiatry, Psychology & Neuroscience, King’s College London, UK; 4 Qualitative Researcher, Nuffield Department of Primary Care Health Sciences, University of Oxford, UK; 5 Lay Research Advisor, Nuffield Department of Primary Care Health Sciences, University of Oxford, UK; 6 Rhodes Trust Professor of Organisational Behaviour, Saïd Business School, University of Oxford, UK; 7 Follow-up Sister in Critical Care, Royal Berkshire NHS Foundation Trust, UK; 8 Senior Research Scientist, Nuffield Department of Population Health, University of Oxford, UK; 9 Chief Executive, Picker Institute Europe, UK; 10 Chief Research Officer, Picker Institute Europe, UK; 11 Programme Coordinator, Nuffield Department of Primary Care Health Sciences, University of Oxford, UK; 12 Associate Professor, Nuffield Department of Primary Care Health Sciences, University of Oxford, UK; 13 Professor of Medical Sociology, Nuffield Department of Primary Care Health Sciences, University of Oxford, UK

**Keywords:** learning community, patient experience data, team-based capital

## Abstract

**Objectives:**

Improving patient experience is widely regarded as a key component of health care quality. However, while a considerable amount of data are collected about patient experience, there are concerns this information is not always used to improve care. This study explored whether and how frontline staff use patient experience data for service improvement.

**Methods:**

We conducted a year-long ethnographic case study evaluation, including 299 hours of observations and 95 interviews, of how frontline staff in six medical wards at different hospital sites in the United Kingdom used patient experience data for improvement.

**Results:**

In every site, staff undertook quality improvement projects using a range of data sources. Teams of health care practitioners and ancillary staff engaged collectively in a process of sense-making using formal and informal sources of patient experience data. While survey data were popular, ‘soft’ intelligence – such as patients’ stories, informal comments and observations – also informed staff’s improvement plans, without always being recognized as data. Teams with staff from different professional backgrounds and grades tended to make more progress than less diverse teams, being able to draw on a wider net of practical, organizational and social resources, support and skills, which we describe as team-based capital.

**Conclusions:**

Organizational recognition, or rejection, of specific forms of patient experience intelligence as ‘data’ affects whether staff feel the data are actionable. Teams combining a diverse range of staff generated higher levels of ‘team-based capital’ for quality improvement than those adopting a single disciplinary approach. This may be a key mechanism for achieving person-centred improvement in health care.

## Introduction

Patient experience – alongside patient safety and clinical effectiveness – is acknowledged as a key component of quality of care.^1^ Whilst there has been considerable focus in several health systems on developing measures and collecting data on patient experience, there remains a gap between measurement and improvement. Collecting data but not using them to improve care is not only wasteful but arguably unethical.^2^ Although some hospitals in the United Kingdom (UK) now have a designated ‘Patient Experience Office’, the office’s function is often primarily data collection and reporting rather than quality improvement, which may be led by a different department in the organization.

Previous studies examining how patient experience data are used for improvement have focussed on board or whole-organization level, and have often reported that interest in using experience data is not always matched by action or the skills to work with such data.^3,4^ Gleeson et al.’s^5^ international systematic review of approaches to using patient experience data for quality improvement in health care confirms that quantitative surveys remain the most common form of patient experience data, and that qualitative data are more difficult to use in terms of time and expertise. Gleeson et al. note that patient experience-based improvement remains focussed on small incremental service changes, which do not require infrastructural or clinical-behaviour change.

There is some evidence of promising frontline approaches, including facilitated feedback of survey findings to ward teams^6^ and experience-based co-design,^5,7,8^ using narrative and observational data. Sheard et al.’s ‘Patient Feedback Response Framework’ focusses on ward teams’ engagement with patient feedback^9^ and identifies three components needed to effect change: normative legitimacy (staff’s personal belief in the importance of responding to feedback and desire to act); structural legitimacy (staff’s perception of sufficient ownership, autonomy and resources needed to establish a plan of action); and organizational readiness (senior hospital management/organizational support for the team to work on improvement, and capacity for interdepartmental collaboration).

Recurring themes in the literature are the misalignment of managerial expectations with how engaged and supported frontline staff feel to make improvements, along with issues concerning the way staff feel about the nature of the data.^3,10–13^ Flott et al.^3^ outline a familiar set of challenges in using survey feedback at clinical level, including scepticism about data quality; lack of training in social research methods; isolated data not linked to other relevant data sources; statistical complexity and lack of technical guidance; and aggregation of data at National Health Service (NHS) Trust (i.e. institutional) level, which does not inspire local clinical ownership or inform ward-level improvements. More recently, Sheard et al.^13^ report that frontline staff struggle with ‘the function and usefulness of the individual data sources’^13(p4)^ and that, while managers recognize this, they seem ‘powerless to prevent the tsunami of ongoing data collection.’^13(p6)^

Martin et al.^14^ outline the value of ‘soft’ intelligence – insights from qualitative and informal sources that might improve care. Despite recognizing its value, staff often do not feel confident to interpret or act on such data, while formal, managerial processes designed to render it useful and credible (including aggregation and triangulation) may inadvertently silence it at source.^15^

### Gaps in the evidence base

Two trends in the study of how patient experience data are used for quality improvement can be identified. First, the meaning of ‘data’ is often treated as a given, rather than examining what staff do in practice with the information they have and whether this information constitutes data. While ‘data’ are not always defined, they are assumed to encompass formally collected and organizationally sanctioned information, particularly from national and local bespoke surveys. Other sources of data have been less well investigated, leaving a knowledge gap as to what such data are or how they are marshalled by frontline staff in their day-to-day work. Without official organizational sanction, potentially useful intelligence may be disregarded.^16,17^

Second, studies of how data are used have focussed on contextual and attitudinal factors, but there has been less consideration of how team constitution affects the type and success of improvements undertaken.

In both respects, a focus is needed on the practical undertaking of quality improvement by frontline staff – that is, an orientation to what people do in practice rather than theories of change or post hoc accounts. In this ethnographic study, we documented how frontline hospital ward teams engaged with patient experience data when encouraged to do so, what challenges they faced and how they could be better supported to work on person-centred quality improvement.

## Methods

Between July 2016 and July 2017, we undertook a case study evaluation in six hospital sites in England’s NHS, as part of a study commissioned by the UK National Institute for Health Research.^18^

A lay panel of 10 people with recent personal or family experience of inpatient care, chaired by lay coinvestigator and coauthor JB, met regularly with the research group to reflect on and make sense of evolving findings.

### Focussed team ethnography

Adopting a longitudinal design,^19,20^ three ethnographers (SP, CM and AC) collected various data from stakeholders at each site to understand how they used patient experience data, including observational fieldnotes, interviews, documents and photographs. Team ethnography is not uncommon in organizational research, but poses challenges and risks,^21,22^ given that no single ethnographer has detailed familiarity with all the sites and individual ethnographers’ interpretations may be difficult to reconcile or synthesize. This required deliberate strategies to communicate regularly, coordinate data collection and ensure analysis was shared and synthesized.

Among its advantages, team ethnography brings multiple lenses and professional experience to bear. The ethnographers had higher degrees in sociology and psychology, and diverse previous research experience. Schlesinger et al.^23^ describe such team-based reflexivity as a positive model which ‘sets up a deliberative process that involves testing the work as it is being done.’^23(p66)^ A team-based approach also enables a greater range, volume and complexity of work within the limited timespan of available funding. Team ethnography therefore enabled us to undertake focussed ethnography across six sites simultaneously. This provided a rapid and condensed alternative to lengthy immersion in conventional ethnography,^24–26^ with shorter, purposive site visits targeted around specific events or meetings.

### Selecting and supporting the sites

Case study sites were purposively selected using a sampling frame derived from analysis of existing national UK survey data and a new survey of NHS hospital patient experience leads. Sites were selected to reflect a range of contexts, from hospitals that were performing well on routinely collected staff and patient experience measures and had a strong track record on quality improvement to those facing organizational challenges and where person-centred improvement was less embedded. Each site nominated a medical ward for the study. A team of staff from each ward took part in a two-day learning community and led their local improvement work. The sites were encouraged to include frontline staff in the core team but were ultimately free to decide on team composition. The learning community met three times during the project. At the first meeting, teams learnt about and discussed with the researchers and lay panel members different types of data such as surveys, narratives/interview data, observations, complaints and online feedback, and approaches to improving patient experience. On returning to their workplace, each team developed and implemented their own ideas for person-centred quality improvement. At the second learning community meeting, they presented their work and the researchers provided formative feedback. At the final meeting, the teams shared their experiences and showcased their achievements with the researchers and invited senior managers from the NHS trusts involved in the study.

### Data collection and analysis

The three ethnographers observed the teams over one year and conducted interviews at the start, middle and end of the fieldwork period. They observed learning community events, local quality improvement planning meetings, meetings of patient and carer experience groups, general staff meetings and workspaces, supplemented by informal conversations with staff. Data collected included written fieldnotes, documents and photographs. The exact nature and amount of observation varied by site, depending on frontline staff’s chosen improvement activities, and was affected by severe workload pressures in the NHS (termed ‘organizational distress’ by one participant) during winter 2016–2017. The pressure on frontline staff to maintain services meant fieldwork was sometimes difficult to arrange or had to be cancelled at short notice. Table 1 summarizes number of visits and hours of observation per site (including learning communities). Not captured here is the amount of time spent emailing, texting and phoning to arrange fieldwork, unquantified activities, which were nonetheless important in building rapport and getting to know and understand teams.

**Table 1.** Observations per site.

	Number of site visits	Total hours observation
Site 1	7	45
Site 2	8	48
Site 3	8	54
Site 4	12	58
Site 5	8	48
Site 6	8	46
Total	51	299

Ninety-five in-depth, audio-recorded interviews with frontline staff and senior managers were undertaken (Table 2), using a shared interview guide. Again, winter pressures created difficulties arranging interviews, particularly at the mid-point of our study.

**Table 2.** Interviews conducted by site.

	Core team interview Time point 1	Core team interview Time point 2	Core team interview Time point 3	Senior/other interviews	Total interviews
Site 1	4	0	4	10	18
Site 2	6	2	5	5	18
Site 3	7	5	3	3	18
Site 4	5	2	2	2	11
Site 5	5	0	3	5	13
Site 6	5	2	4	6	17
Total	32	11	21	31	95

Qualitative data were analysed iteratively by the three ethnographers using an inductively generated coding framework in NVivo 10/11. This included types of patient experience data used, attitudes towards/understanding of data, team composition and membership, relationships with the Patient Experience Office and senior management, and organizational pressures and constraints. As part of the research group’s ethnography design, the group met regularly with the Principal Investigator (PI) during the fieldwork to ensure comparability of data collected and to discuss emerging analysis, developing theoretical explanations as the work progressed. ‘Thick’ case descriptions (incorporating rich contextual detail and interpretation) were produced for each site, along with process maps of each team’s quality improvement projects, as part of a thematic analysis. In line with the protocol, all sites are anonymized to ensure participants felt comfortable sharing more negative views and experiences.

By the end of the project, teams in every site had undertaken improvement initiatives using a variety of data sources. In some cases, this was a single intervention, while in others it involved multiple interlinked projects on a suite of different topics. Our aim in this study was not to quantify change or rank sites in terms of ‘success’ – given that sites were selected deliberately to represent a range of starting points and levels of maturity in working on patient experience, variation in progress was expected. However, some teams undoubtedly struggled more than others. Our focus was rather on a comparative understanding of how staff approached their projects on patient experience, why some struggled and how they made sense of the task.

Below we present an overview of findings on two key themes: the meaning of patient experience data and team-based capital.

## Results

### The meaning of patient experience data

We observed teams engaging in a process of sense-making, drawing on a spectrum of formal and informal sources of intelligence. However, it was not initially obvious to frontline staff what patient experience ‘data’ were, where to locate them in the organization or how to use them. Surveys were the most recognized source of information; however, available survey data were commonly not ward-specific and therefore less useful for frontline quality improvement.

Qualitative data – such as patient stories and free text feedback at the end of surveys – were appealing to staff, seen as convincing evidence and reflecting real experience, but remained under-utilized due to lack of confidence in how they could be used. Few staff had considered observation or shadowing as a source of data until introduced to this by the research group at the first learning community event. Some teams successfully went on to experiment with observations. Online feedback on social media attracted initial interest, but lack of organizational support to access and use it at ward-level prevented team engagement.

The emotional response that patient experience data elicited in staff was evident. Staff were receptive to and motivated by positive feedback from patients, but did not tend to see this as a source of ideas for quality improvement. Negative feedback was reported to be useful, but in practice could be challenging or difficult to accept for staff and, depending on the form it took, inhibited efforts at improvement.

At times, staff were unable to point to a specific piece of patient experience data that led to a particular improvement project. As one consultant said, ‘[The quality improvement intervention] fits right, but I don’t think they’ve done a survey.’ The ethnographer’s accompanying coding note observed, ‘[This] sums up what I think is happening with a lot of the teams … the issues are known to staff and they are responding, without being able to identify any specific piece of patient feedback.’

In another case, a staff member commented, ‘I hadn’t actually heard of patient experience until I went to the learning community.’ This demonstrated the disjunction between patient experience as an organizational and policy construct on the one hand and daily practice on the other.

Sometimes staff reported acting on what they felt they already knew needed changing. This knowledge might come from caring for and observing people, or conversations with carers or colleagues. Taking part in the project gave them a platform to pursue existing concerns and ideas. In some cases, such staff-initiated improvements were backed up by formal patient experience data. This is demonstrated in an interview with a ward manager:

Ward manager: You need to remember that the staff are doing a job all the time. They have ideas that might improve the service … 

Interviewer: Things like the hearing aid boxes I think came from the staff suggestion board, didn’t it, rather than from the patient experience data?

Ward manager: Yeah, all of that just came up because we could see the distress … [The ward administrator] deals with a lot of the lost property, the distress of the patients that have lost an expensive hearing aid and they can’t communicate at a time when they most need to communicate clearly … .That came from, you know, our own experience of what causes them distress.

Some teams explicitly chose to focus on improving staff experience as a route to improving patient experience. The focus was both on using staff experience to understand patient experience and as a legitimate target for action in its own right, on the basis that – to employ an aphorism used at several of the sites – ‘Happier staff means happier patients.’

As the project progressed, those involved in the research – both as participants and as researchers – began to work with an expanded notion of patient experience data. One patient experience officer described this as ‘just general conversations with patients’, encompassing tacit, embodied experience and fleeting observations during daily practice. The officer went on to explain:

It depends how we look at data. I think in the ward staff before, if you said to them, ‘What’s patient experience data?’ they will say, ‘Surveys.’ I’m saying to them now, ‘Data is any feedback at all, wherever that’s coming from and in whatever form, whether that’s coming from focus groups from the patients or anecdotal feedback from staff and patients, it’s all patient experience data.’

In learning community events held as part of the study, teams discussed the potential to see thank you cards, gifts of chocolates or patients giving staff a hug when discharged as tangible expressions of positive patient experience, even though these are rarely recognized as such, apart from – in some cases – a simple count. As one consultant said:

Ward managers are asked to keep a log of any compliments, and then – it sounds really crass – boxes of chocolates – count the number of boxes of chocolates you have and thank you cards.

While staff had experiential insight through their close interactions with patients, it was also acknowledged that staff perceptions may not always be a reliable guide. As a matron noted:

The challenge is there are several, many staff who work within the Trust who will be completely unable to see the impact of their behaviour or their – let’s say – their brusqueness … So that’s a real fine balance, isn’t it? ‘Cos there are people who won’t get that at all. They won’t see that that’s bad patient experience.

The challenge is to find ways to encourage staff empathy and creativity as a route to patient experience improvement without reverting to assumptions that staff know best.

### Team composition and resources

The composition of the frontline teams who took part in the project was determined locally. Each consisted of up to five core individuals. When we recruited sites, we encouraged them to identify a core team of frontline staff (and patients) for the first learning community. Teams interpreted ‘frontline’ differently. Some included from the outset a staff member from their local Patient Experience Office who was assigned to work closely with them on the project. Others brought a ward-only team to the event but liaised closely with the Patient Experience Office during the project. Despite an emphasis at learning community events on mapping key stakeholders and sources of support, a few teams maintained a more distant relationship with their in-house patient experience team. Team composition thus ranged from entirely nursing-led to a mix of nurses, doctors, unqualified nursing assistants, ward administrators, allied health professionals and staff from the hospital’s Patient Experience Office (see Table 3). The disciplinary and pay-grade mix of each team varied considerably, suggesting that there wasn’t an accepted home for patient experience improvement within these organizations.

**Table 3.** Team composition and quality improvement projects.

	Team composition	Improvement projects	Types of data used
Site 1	Core team: three health care assistants and two nurses; occasional support from consultant. No patient experience team involvement.	One quality improvement project: introduction of a welcome pack, well received by patients.	No use of formal existing or new data. Project inspired by staff observation of patient need.
Site 2	Core team: ward nursing manager, ward clerk, health care assistant and member of the patient experience team. Ongoing support from matron and initially from consultant.	Initiatives ranged from small patient-focused interventions to ward-wide initiatives to facilitate a culture of open communication with patients. These included colourful hearing aid boxes for patients to place their hearing aids into so as not to lose them; admission packs; provision of information boards and leaflets; interventions to encourage communication and understanding between patients and staff, such as ‘It’s OK to ask’ badges and posters, bedside whiteboards and a ‘What matters to me’ board for patient and staff comments.	Wide range of data sources including ward survey, patient focus group, ‘What matters to me’ comments tree, staff-generated ideas. Existing patient experience data (national inpatient survey data, Friends and Family Test) data drawn on to provide corroboration of ideas from other sources.
Site 3	Core team: ward nursing manager, activities and well-being co-ordinator (health care assistant), head of quality improvement and patient experience and a patient experience officer. Peripheral membership included nursing staff and an executive director with extensive experience in quality improvement.	Experience-based co-design project identified three areas of action/improvement: improving communication, increasing responsiveness to patients’ needs and increasing interaction with patients. Several interventions were implemented to address each of these issues, including the design of a welcome pack for new patients and a discharge pack, a photo board to help patients/relatives identify staff (and their role), call-bell use and response times, socialized dining in the social room, introduction of a structured activities timetable and bespoke activities, increased one-to-one time with the activities and wellbeing coordinator.	Additional questions added to Trust’s own real-time survey for this project. Experience-based co-design methods, including group interviews/discussion with patients and observations on the ward.
Site 4	Core team: ward manager, nursing staff, senior health care assistant, patient experience project manager. During the project, other staff were integrated into the team including a pharmacist, medical registrar and charge nurse, as well as peripheral participation by at least five junior doctors.	Main focus on provision of information and more efficient operational processes, especially discharge. Activities included the ambitious redesign of the discharge process (including prescribing) to be more efficient, following a series of successful stakeholder mapping sessions; and the design of information leaflets for patients, covering the ward’s most frequent conditions.	Ward survey identified initial priority area. Additional experimentation with ward observations, patient diaries, and patient interviews after discharge.
Site 5	Core team: ward manager, two nurses and two health care assistants. Initial input from a patient representative.	Strong focus on changing culture and nature of interaction. Numerous small-scale changes, e.g. sending sympathy cards to bereaved relatives; promoting (and auditing the use of) sleep-well packs to address noise at night; having nurses accompany doctors on ward rounds, to help with patient understanding and communication; mainstreaming patient experience into routine ward practices (e.g. patient experience featuring in induction for new staff and having a regular slot in staff meetings).	Team felt existing data did not give much insight, so gathered new data through inviting in a colleague from Professional Development to observe ward care and conduct staff interviews exploring their views on patient experience. Data from patients collected on feedback board.
Site 6	Core team: Two quality nurses, matron, ward nursing manager and associate chief nurse for medicine. A patient representative was involved in the first learning community and regularly attended project meetings. The patient representative was influential in eliciting initial focus of the team’s collective project.	Focus was on improving staff morale and communication, on the basis that improved staff experience would lead to better patient experience. One aim was to re-organize communication at shift handover, including more focussed discussions at the end and beginning of shifts; ward-based ‘It’s OK to ask’ scheme (to improve patient-staff communication); and a trial run of sleep packs for patients. Other reported initiatives tended to be Trust-wide and centrally rather than locally driven, and many of the team’s original plans were not fulfilled at the end of fieldwork.	Ward staff survey. Some observations of ward care. Sleep pack was inspired by shared ideas at the learning community, rather than local patient experience data.

Teams consisting of a variety of different professionals tended to make more progress with their chosen quality improvement projects and work on a wider range of topics. Staff did not necessarily need to be senior or from a particular profession to effect change: in one team, a ward administrator was pivotal in making practical changes; in two sites, nursing assistants played important roles; in another, the dynamic and focus of the project changed as a pharmacist and junior doctors in training became involved as peripheral team members. By contrast, one team, which consisted entirely of relatively senior nursing staff, did not seem to make much progress at ward level. In addition, those teams with a close relationship to the Patient Experience Office tended to be more ambitious in the improvement activities addressed. Patient Experience Office staff were well positioned to provide data (and the skills to interpret it) and act as project managers, variously sourcing materials, generating resources, taking plans forward and maintaining momentum.

‘Space’ was a recurring theme: physical space in terms of places to meet, and figurative space – space in the day amidst heavy workloads and shift patterns with limited handover times, and cognitive capacity to take on projects (or ‘head space’ as one person described it). Below, a senior sister explains how resources provided by a patient experience officer increased team capacity:

If you look at my job description, improving is obviously something that’s in there. But I’ve got a full-time job that doesn’t really give me any additional time to take on a big project … So you do need a project manager that’s got the time and resource and that’s her job … It’s a relief that I’ve got someone else in the background, because I know if it was left to me to organize meetings it probably wouldn’t get very far.

In another example of blending resources, a team of nurses and nursing assistants called on a consultant with greater institutional connections and authority, but who was not part of the core team, to help them apply for funding. The consultant explained:

All I’ve done is the paperwork to say, ‘This is what we want to do, and we want £1000 please.’ … I’ve done loads of bids before, usually for equipment and, yeah, I know how to do it, and I think they looked at it and thought, ‘Oh God, this is going to take us hours,’ but in fact it doesn’t. You know, it took me half an hour …  I suppose the nurses would struggle with that because they don’t know these people, and that’s something that I could just do at the click of a button.

Teams with fewer collective social and organizational resources – e.g. those with a more restricted range of staff involved, and those with more distant relationships with the Patient Experience Office – faced greater challenges. In a few cases, teams seemed to actively resist forming relationships with the Patient Experience Office, preferring to rely on strong internal, single disciplinary bonds, but, as a result, missing out on skills and wider organizational endorsement.

Restricted team membership could be compounded by other practical resource challenges. This could be as simple as having no quiet space to meet. One ethnographer’s fieldnote mentioned:

[The team member] left her room and took us to the family room, but noting that it was now occupied by junior doctors in meeting, she decided we would have the meeting in the reception area. En route to the reception area, she spotted an empty single bedroom (approx. 8ft by 4ft room) and decided to hold the meeting there.

Another challenge was having no sustainable funds for even minor improvements, such as copying leaflets. As a nursing assistant explained:

We have found hurdles all of the time … Trying to get money to start with. You know, we were phoning up in our own time, phoning people, companies – ‘Can you do this for such a price? Can you do this for such a price?’ … So, we were bartering all of the time.

## Discussion

The formation of teams of people from different disciplines and grades established a network of individuals with assorted levels of Bourdieu’s^27^ four forms of capital: economic, social, symbolic and cultural (see Table 4). We suggest this is a key mechanism for achieving person-centred improvement in the NHS.

**Table 4.** Capital in the NHS.

Type of capital	Examples in the NHS
Economic	Infrastructure, including office/ward equipment, meeting space, staff capacity and time, materials.
Social	Access to networks and alliances across different clinical and non-clinical departments.
Symbolic	Reputation and status within a given setting, influencing ability to recruit, delegate and move agendas forward.
Cultural	Knowledge/expertise in particular forms of practice – e.g. medical conditions, administration, patient experience.

NHS: National Health Service.

Team-based capital operates both through the differing knowledge and skills individual team members bring, and through their ability to generate varied practical, organizational and social resources for patient-centred quality improvement. While organizational rhetoric stresses the value of good patient experience, in reality it may still have lower organizational priority than patient safety and clinical effectiveness.^2^ A coalition of people and resources is important to challenge this fragile status and raise the profile of patient experience work.

Linked to this is the question of what counts as patient experience data, and what is formally sanctioned by the organization as important and actionable. A key finding from this work is the expanded notion of ‘data’. We question whether data have to come directly *from* patients to constitute ‘patient experience data’, and for expressions or understandings of patient experience to be formally recorded or documented to count. In her work on patient experience in mental health units, Pols^28^ noted that nurses’ intuitive understanding from daily practice was a valuable additional insight alongside interviews. Nurses, she argued, ‘attend to what silent patients like or dislike … They seem to know what individuals prefer, and if they do not, they try to find out by trial and error.’^28(p210)^ At the same time, she acknowledged the risk that ‘there are situations in which a conflict is clear, and in which nurses do not take the appreciations of patients into account or overrule them.’^28(p217)^ This may have a parallel in the patient safety field with the concept of ‘exnovation’ – understanding how good safety is often accomplished through staff’s existing, taken-for-granted practices or ‘hidden competence’, rather than explicit new innovations.^29^

Staff with the least power in the organization – unqualified nursing assistants, ward administrators, cleaners and porters – may be rich in this kind of tacit, embodied intelligence but are unlikely to be able to mobilize such knowledge for improvement without wider team capital behind them. This resonates with Sheard et al.’s account of structural legitimacy (whether staff feel they have sufficient ownership, autonomy and resources to act on patient experience data).^9^

## Limitations

Our findings are derived from only six UK hospital sites, but nevertheless provide rich data relevant for other contexts both nationally and internationally. We did not set out to demonstrate whether particular types of patient experience data or approaches to quality improvement are more effective than others, and we are therefore unable to draw conclusions on this point.

## Conclusion

We suggest that frontline ward teams, as well as senior managers, need support in recognizing and harnessing a wider range of patient experience data for health care improvement, and to understand where such data reside within the organization. This signals a shift from top-down measurement for performance to an approach which embraces frontline wisdom and creativity, and involves a broad coalition of staff in improvement work.

We recommend particularly that those leading patient experience work in hospitals actively seek to include not just authoritative senior figures, but also hands-on frontline staff, whose perspective too often remains unvoiced and unnoticed. Patient Experience Office staff themselves also need support in engaging with multiple forms of data. As one contribution to this, we have worked with the Point of Care Foundation, an independent charity providing evidence and resources to support health care in the UK.^30^ We have produced an open-access guide to using patient experience for improvement, aimed at frontline staff and patient experience teams and incorporated into face-to-face training courses led by the Foundation.^31^
